# Supplementary material for: Direct and integrating sampling in terahertz receivers from wafer-scalable InAs nanowires
Source: Nat Commun. 2024 Jan 2;15:103. doi: 10.1038/s41467-023-44345-1 (PMC10761983; doi:10.1038/s41467-023-44345-1)
Supplement: Supplementary file 1 — Supplementary Information [file 41467_2023_44345_MOESM1_ESM.pdf]

Supplementary Information for

Direct and Integrating Sampling in Terahertz Receivers from Wafer-Scalable InAs Nanowires

Kun Peng, Nicholas Paul Morgan, Ford Wagner, Thomas Siday, Chelsea Qiushi Xia, Didem Dede, Victor Boureau, Valerio Piazza, Anna Fontcuberta i Morral, Michael B Johnston

This PDF file includes:

1. Finite-difference time-domain (FDTD) simulation
2. Fourier transform infrared (FTIR) photocurrent spectroscopy
3. Terahertz (THz) time-domain spectroscopy system
4. Sampling modes for photoconductive THz detection
5. Excitation wavelength-dependent THz detection
6. High-resolution scanning transmission electron microscopy (HR-STEM) investigation
7. Other THz measurements on InAs nanowire receivers
8. Side-facet tuning for multi-faceted InAs nanowires

Figures S1 to S11

Table S1

1. FDTD simulation

Commercial software (Ansys-Lumerical) based on the finite-difference time-domain (FDTD) method was used to perform simulations in the THz range to examine the influence of the modification of antenna geometry. The FDTD simulations were set up using the geometries shown in Figure S1. In order to make the simulation environment close to the experimental conditions, a broadband pulsed Gaussian Source (with a beam diameter of 3 mm) was used as the simulation source with characteristics (such as pulse profile and bandwidth) imported from experimental data measured using the ZnTe crystal receiver. A time monitor (point) was placed at the centre of the bowtie antenna to measure the incident THz pulse in the time domain. All experiments and simulations were performed at normal incidence, and the polarisation of the incident THz electric field was aligned parallel to the nanowire (NW) length axis (as used experimentally). Owing to the large and negative real part of the relative permittivity of most metals at THz frequencies, the perfect electrical conductor material model was employed for simulations. In contrast to the experimental gold thickness of 140 nm, a thickness of 4μm was chosen in order to avoid too small a mesh size in the z direction. A mesh override region was added to the gold layer to force a 2 μm mesh (dx=dy=dz= 2 μm) in simulation.

Figure S1(a) show FDTD simulations of local THz electric field enhancement at the bowtie antenna's gap region of different faced tip-to-tip profiles (at 1 THz). The modified round-tip profile leads to a maximum field enhancement of 80 times and a rather uniform field enhancement across the whole gap when compared to that of the original point-tip profile. FDTD simulations also confirm that the change of the faced tip-to-tip profile in the bowtie antenna have no influence on the antenna's spectral response to the incident THz signal.

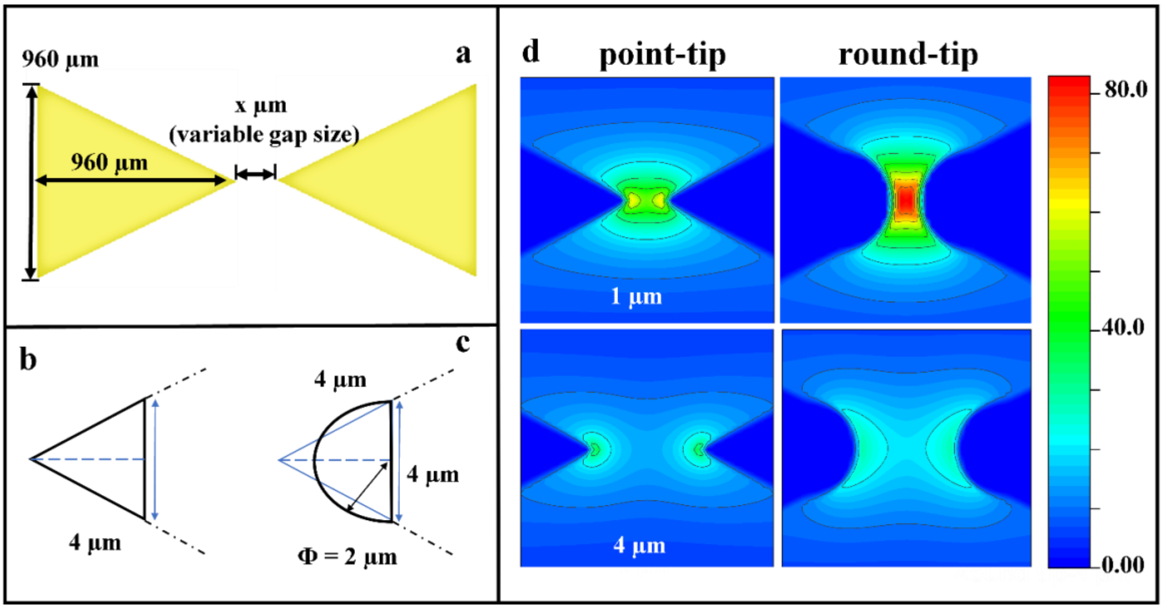

Figure S1. Antenna geometry. (a) Schematic representation of the bowtie antenna structure used in this work. (b) The faced tip-to-tip profile in the original bowtie antenna. (c) The modified tip-to-tip profile in the bowtie antenna for experimental use. (d) Comparison of the FDTD simulated THz electrical field distribution at the gap region of the bowtie antenna (at 1 THz), where the left column is the case of using point-tip corresponding to (b), the right column is the case of using round-tip corresponding to (c), the top row is the case when gap size is at 1 μm and the bottom row is the case when the gap size is at 4 μm.

2. Fourier transform infrared photocurrent spectroscopy

The spectral photoresponses of InAs NW receivers were investigated using a Bruker Vertex 80v Fourier transform infrared (FTIR) spectrometer at room temperature under vacuum, as shown in Figure S2. The FTIR spectrometer was utilised as a light source and interferometer, and the NW receiver connected to an ultra-low noise voltage source and current preamplifier. The output of the preamplifier was digitised by the FTIR's analogue-to-digital converter and then the received interferogram was Fourier transformed from the time domain into the frequency domain using a computer controlled by Bruker's spectroscopy software OPUS. All spectra were scanned with 0.158 cm/s scan velocity. The spectrometer was equipped with a CaF_2 beam splitter combined with white light (tungsten lamp 1000 - 25000 cm^{-1}) and KBr beam splitter combined with IR light source (globar 10 - 13000 cm^{-1}) for near- and mid- infrared region measurements, respectively. Considering the spectrum of the light source and the efficiency of the beam splitter in the spectrum, a spectral response correction was performed on all the measured data. A commercial InGaAs photodiode is used as reference to demonstrate acceptable validity and reliability of the correction.

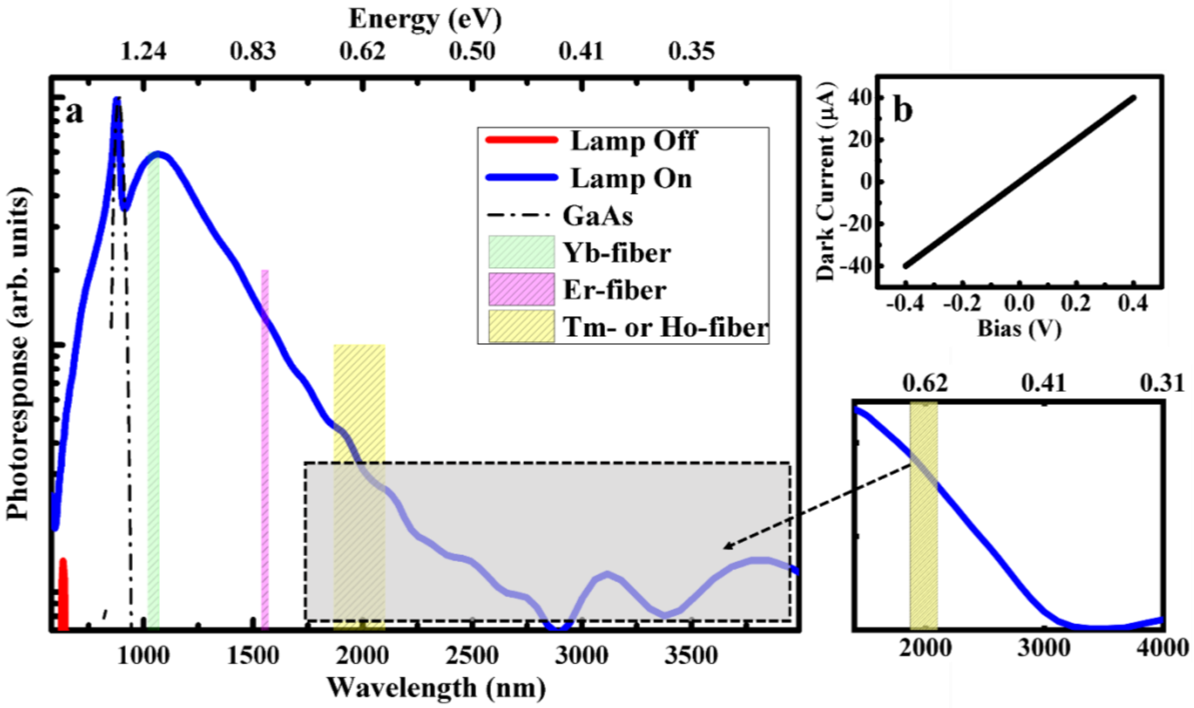

Figure S2. FTIR photocurrent spectroscopy. (a) Semi-log plot of photocurrent spectral responses from a 20NW InAs receiver measured by FTIR. Left: under white light source (tungsten lamp) combined with CaF_2 beam splitter. Right: under globar source combined with KBr beam splitter. The grey dashed square indicates the spectral range where the measured data is more appropriate when using globar source as shown in the right small figure. (b) Typical dark I-V characteristics of a 5NW InAs receiver, showing a linear current-voltage curve, thus confirming the formation of ohmic contacts to the InAs NW receiver.

We have added the centre wavelengths of currently available fibre-based femtosecond lasers as coloured bars in the photocurrent spectrum (see Figure S2). It is clear that the NW photoconductors are suitable for operation with Yb:fibre (~1.03 μm), Er:fibre (~1.55 μm) and Tm-/Hm-fibre (~2 μm) lasers.

A sharp photoresponse peak located around 870 nm has been correlated to the leakage photocurrents from the zincblende GaAs substrate that has a band edge energy of ~1.42 eV at room temperature. This was confirmed by a photocurrent measurement of a reference receiver without NWs and nanoridges grown on it, indicating measurable tunnelling and/or shunting currents through the 25-nm SiO_2 layer between the GaAs substrate and gold bowtie electrodes. Nevertheless, the leakage currents become negligible for illumination with photon energies smaller than the GaAs bandgap energy (< 1.42 eV, equivalently > 900 nm), which is the case for photoconductive THz receivers gated with telecom-wavelength lasers (1.2 - 1.6 μm).

3. THz time-domain spectroscopy system

As described in Methods, a regenerative laser amplified system (Spectra Physics, MaiTai - Empower - Spitfire) with an average power of 4 W, central wavelength of 800 nm, pulse duration of 35 fs and repetition rate of 5 kHz was used. The laser beam was split into two beam paths: one was guided to a spintronic THz emitter to generate a single-cycle linearly-polarised THz pulse in the system; the other, going through an optical parametric amplifier (TOPAS, Light Conversion) to allow wavelength tuning from 290 nm to 2.6 μm, was guided to the receiver to generate photocarriers. A schematic of the THz time-domain spectroscopy setup is presented in Figure S4. The THz pulse was directly detected by the NW receivers with the THz polarisation direction aligned with the NW length axis (that is the gap orientation). All measurements were repeated at least 3 times, from which the uncertainty was determined by the standard deviation.

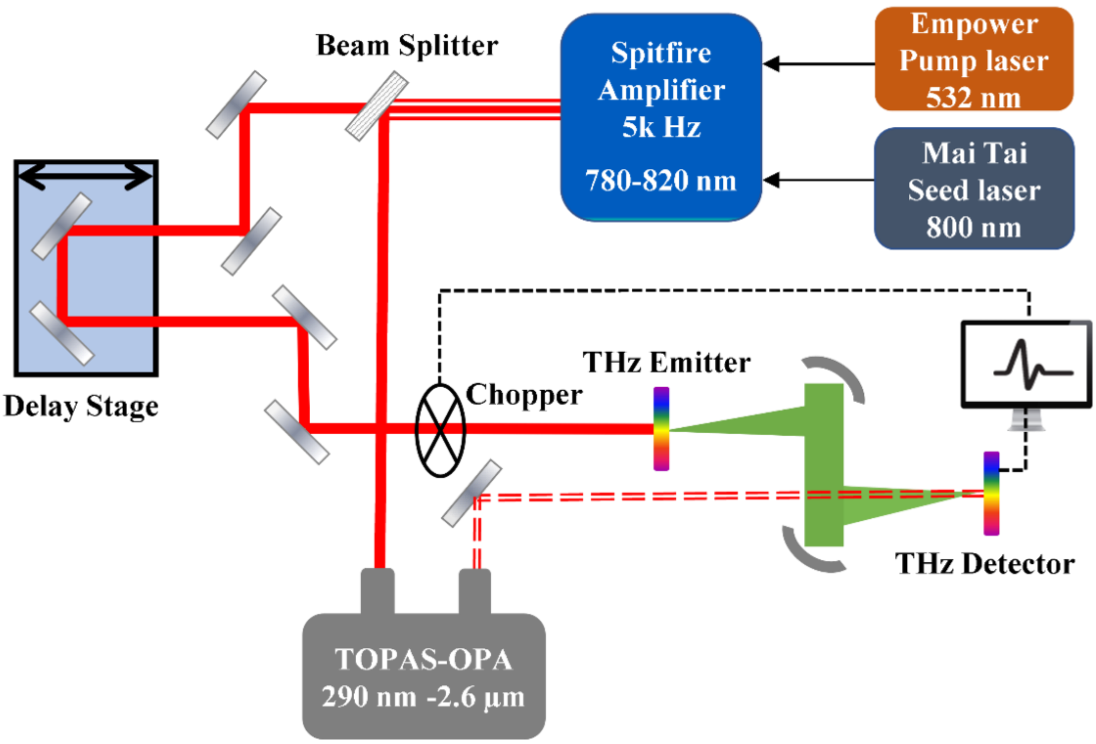

Figure S3. THz system. Schematic of the custom-built THz time-domain spectroscopy system utilised in experiments.

4. Sampling modes for photoconductive THz detection

As described in ref. 1, the measured current transient relates to the electric field of the incident THz pulse depends on the type of photoconductive receiver (which determines the sampling mode), and is expressed by

$$I(\tau) \propto \int_{-\infty}^{+\infty} E_{(THz)}(t)\, \sigma(t-\tau) dt \qquad (1)$$

where τ is the time delay between the THz pulse and the laser pulse, $E_{(THz)}$ is the effective electric field of the THz pulse at the photoconductive receiver and $\sigma(\tau)$ is the time dependant photoconductivity of the detection material. By changing the time delay between the THz pulse and laser pulse (via precise path length control in optical delay line), the temporal profile of the current transient can be mapped out, which can be further processed to recover the THz electric field $E_{(THz)}(t)$. In general, the types of photoconductive receivers can be categorized into three groups, including direct sampling for a photoconductive material for short carrier lifetime (<< 1 ps), convolved sampling for a photoconductive material for a carrier lifetime (1 ps < τ < 100 ps), and integrating sampling for long carrier lifetime (>> 1 ps).

Figure S4 shows the raw data of THz-induced current transients measured from two representative InAs-NW receivers studied in this work and two reference receivers (including a bulk Fe^{+}-implanted InP receiver[1]) and a single-InP-NW receiver[2].

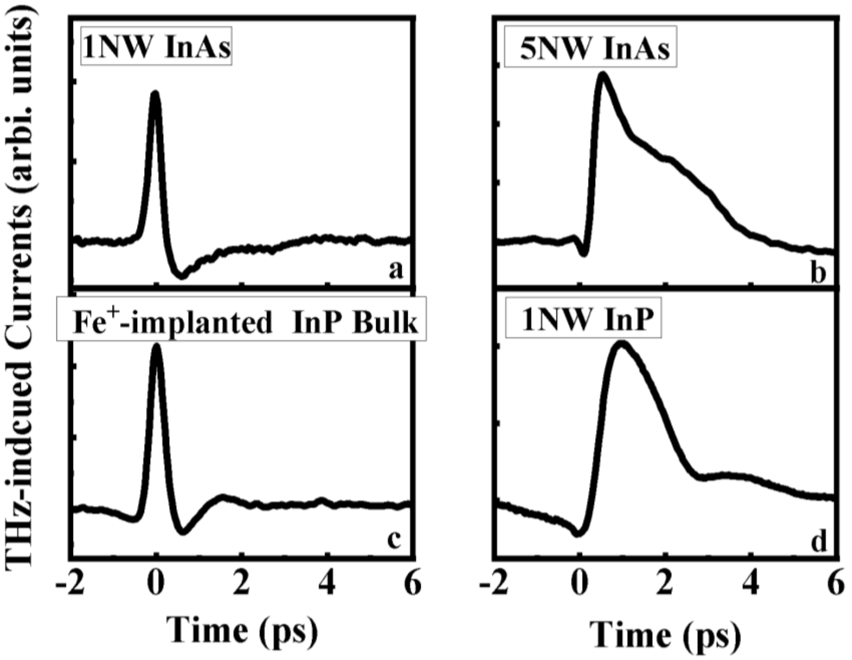

Figure S4. Comparison of THz response. Comparison of raw data of the THz responses measured from (a) a 1NW InAs receiver, (b) a 5NW InAs receiver, (c) a Fe^{+}-implanted InP receiver and (d) a 1NW InP receiver, respectively. Their sampling modes are identified as direct sampling for (a) and (c), and integrating sampling for (b) and (d).

As shown in Figure S4(d), the InP NW has the longest photoconductivity lifetime of ~4 ns and thus its corresponding receiver operates in integrating-sampling mode, which have been reported in our previous work[2]. The (single-faceted) 5NW InAs receiver as shown in Figure S4(b) exhibited a similarity

in its THz response profile to that of a 1NW InP receiver, indicating it operates in integrating sampling mode as well, implying the single-faceted InAs NWs has a long photoconductivity lifetime > 100 ps.

The Fe^{+}-implanted InP bulk has a very short photoconductivity lifetime of ~ 0.4 ps[1] and thus its corresponding THz receiver operates in directing sampling mode as shown in Figure S4(c). The (multi-faceted) 1NW InAs receiver shown in Figure S4(a) exhibited a similarity in its THz response profile to that of a Fe^{+}-implanted InP bulk receiver, indicating it is a direct-sampling-type receiver, suggesting the multi-faceted 1NW has an ultrashort photoconductivity lifetime (< 1 ps).

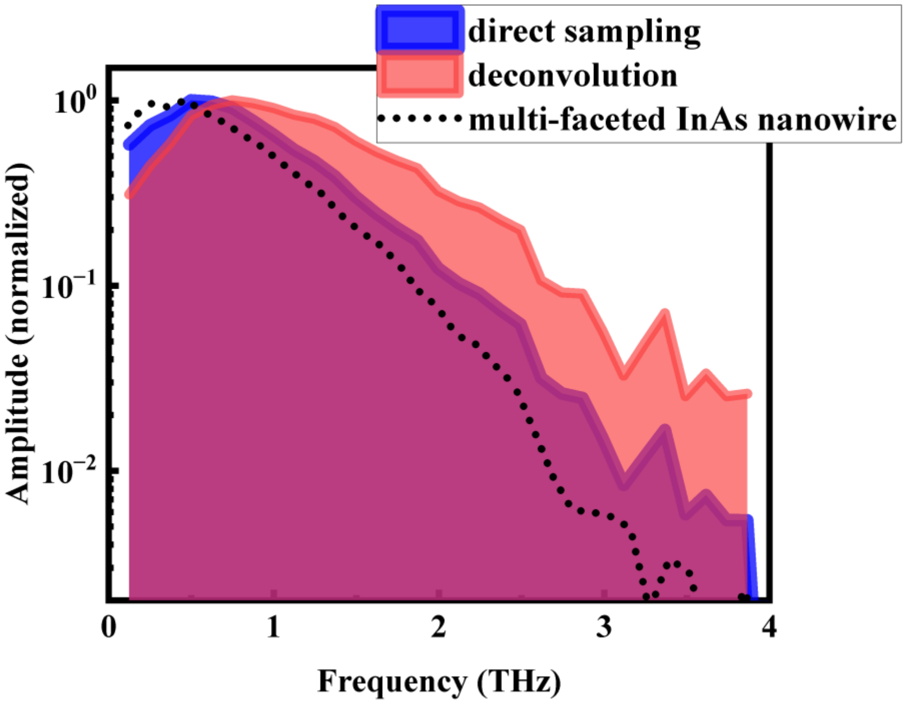

Figure S5. Deconvolution of THz signal. THz spectra calculated by unprocessed THz-induced current measured from a Fe^{+}-ion implanted InP bulk receiver (approximated as a direct-sampling receiver) and deconvolution of the photocurrent data with photoconductivity lifetime of 0.4 ps (regarded as a convolved receiver).

Deep insights into the side facet/facet combination types and their associated surface-recombination-limited carrier lifetime in horizontal InAs NWs need to be investigated. However, the related measurements are rather difficult due to the lack of proper characterisation tools. Mid-infrared micro-photoluminescence measurements have been reported for the study of the dominant recombination mechanisms and surface state densities in InAs NWs[3]. Such an approach, however, only offers insights into their optical properties and has to be performed under low temperature owing to the weak band-edge emission in InAs NWs. Optical-pump THz-probe (OPTP) spectroscopy is the most suitable technique to extract the photoconductivity lifetime and the surface recombination velocity for NWs[4]. However, OPTP measurements are currently based on the investigation of ensemble of NWs. OPTP measurements on individual semiconductor nanostructures is under development in our group, which will be demonstrated in our follow-up work.

Furthermore, in the ideal scenario, direct sampling requires a photoconductive material to have a short carrier lifetime of <<1 ps. In this work, the Fe^{+}-implanted bulk InP with a photoconductivity lifetime of ~ 0.4 ps was used to fabricate a bulk photoconductive THz receiver. Figure S5 shows the THz spectrum recorded by the receiver using the direct sampling approximation and after deconvolution to

account for the finite (0.4 ps) photoconductivity lifetime of the InP. The deconvoluted spectrum is a better representation of the true spectrum of the THz pulse that was incident on the receiver. The direct sampling processing is a reasonable approximation, but underemphasises the higher frequency spectral components. Since the studied multi-faceted InAs 1NW receivers shows a high degree of similarity in terms of their THz responses to the Fe^{+}-implanted InP bulk receiver, this implies that the multi-faceted InAs NWs most likely have a photoconductivity lifetime of a few hundreds of femtoseconds, similar to that of the Fe^{+}-implanted InP bulk.

After confirming the sampling mode of each type of receivers, we processed their raw THz response data accordingly to evaluate the performance of the NW receivers, as shown in Table S1.

Table S1. Detector performance comparison. Comparison of detection performance between 1NW, 5NW and 20NW receivers. (For signal-to-noise ratio of time-domain data, the signal is defined as the peak-to-peak current over one time-domain scan, and the noise is the standard deviation of the difference of two consecutive scans with identical parameters. Dynamic range of time-domain data is defined as the ratio of the peak-to-peak current over one time-domain scan to the standard deviation of the noise current in the absence of THz over the same scan. Dynamic range of spectral data is defined as the ratio of the maximum amplitude to the root mean square of the noise floor of the amplitude spectrum. Spectral response bandwidth is defined as the cut-off frequency at the noise floor of its spectral response.) All NW receivers have an identical antenna gap size of 1 μm and were optically gated with 1.5-μm laser pulses of 1.66 mJ/cm^2 fluence.

		Detector Type		
		Photoconductive Antenna (Bow Tie)		
	Performance	1NW	5 NW	20 NW
	NW Facet Type	Multi-Faceted	Single-Faceted	Single-Faceted
	Sampling Modes	Direct	Integrating	Integrating
Raw Data in Time Domain		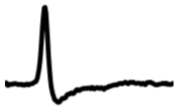	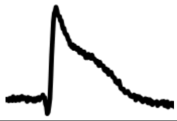	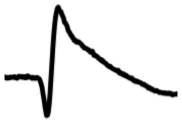
	Signal-to-Noise Ratio	6.0	22	28
	Dynamic Range of Time-Domain Data	94	94	140
Converted Spectral Data in Frequency Domain		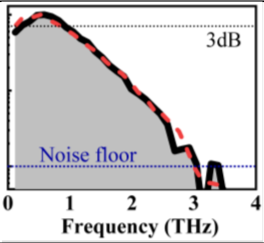	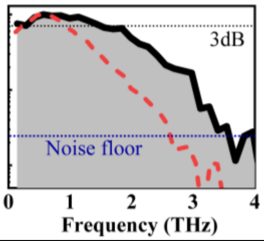	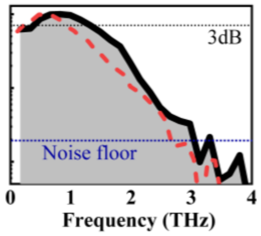
	Dynamic Range of Spectral Data	580	130	230
	Spectral Response Bandwidth (THz)	3.0	3.0	3.0
	3dB Cut-Off Frequencies (THz)	0.14- 0.91	0.14-1.49	0.38-1.34

*Signal-to-noise ratio determines the accuracy and stability of the measurement.
*Dynamic range determines the ability to measure strongly attenuating samples.
*The red dashed line in spectral data represents the original spectrum of THz source measured by a bulk ion-implanted InP receiver.

5. Excitation wavelength-dependent THz detection

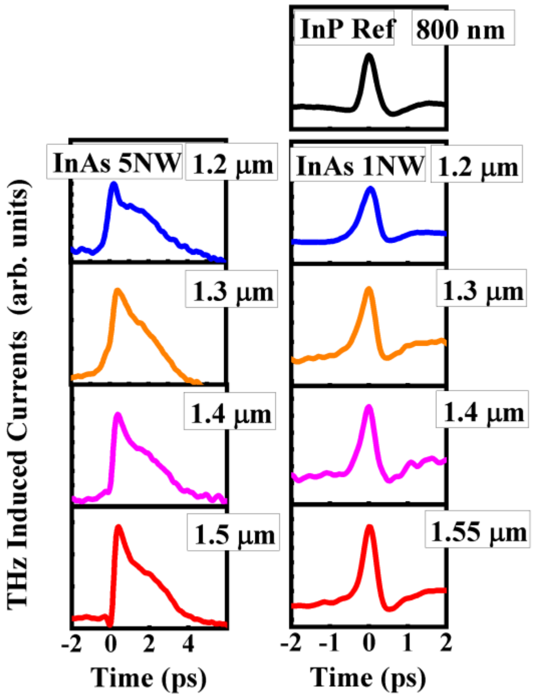

Figure S6. Excitation wavelength investigation. (Left column) Raw data of THz responses obtained from a single-faceted 5NW receiver, optically gated with 1.2-µm, 1.3-µm, 1.4-µm, 1.5-µm laser pulses of 0.98 mJ/cm^2 fluence, respectively. (Right column) Raw data of THz responses obtained from a multi-faceted 1NW receiver, optically gated with 1.2-µm, 1.3-µm, 1.4-µm, 1.55-µm laser pulses of 0.98 mJ/cm^2 fluence, respectively. Note: raw data of THz response obtained from an ion-implanted InP bulk receiver was provided as reference (see black line), which was optically gated with 800-nm laser pulses of 1.5 mJ/cm^2 fluence.

6. High-resolution scanning transmission electron microscopy (HR-STEM) investigation

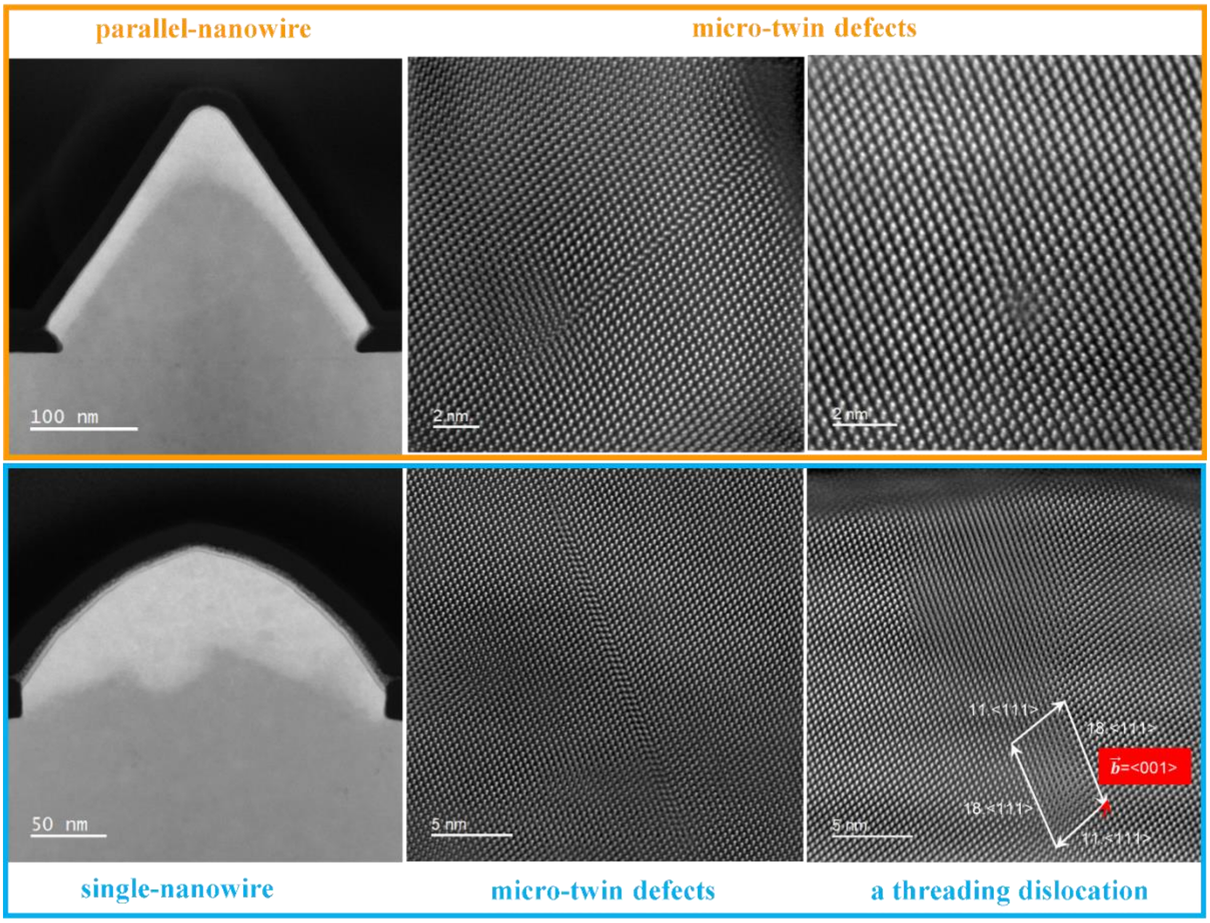

Figure S7. HR-STEM investigation. HAADF-STEM overview and high-resolution micrographs (average background subtraction filtered) of representative NWs studied in this work. Top: From multi-NW array, exhibiting single-faceted morphology. Bottom: From single-NW device, with a NW length of 14 µm, exhibiting multi-faceted morphology. High-resolution images reveal micro-twin and threading dislocation defects inside the respective InAs NWs.

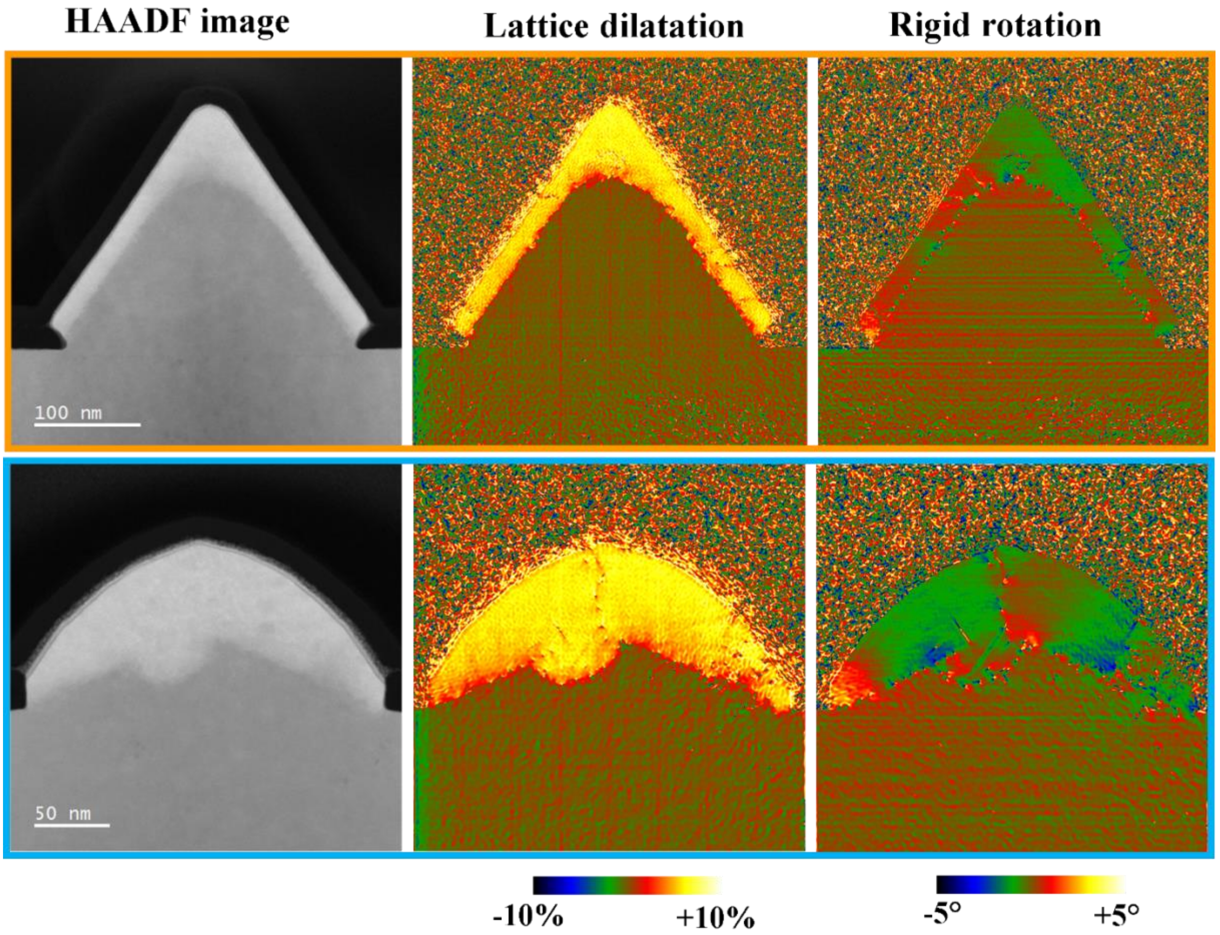

Figure S8. Strain mapping. HAADF-STEM overview and geometric phase analysis[5] showing strain and rotation maps of representative NWs studied in this work. Top: From multi-NW array, exhibiting single-faceted morphology. Bottom: From single-NW device, with a NW length of 14 μm, exhibiting multi-faceted morphology. The (in-plane) lattice dilation map reveals that the InAs NW is relaxed through a network of misfit dislocations at the InAs-GaAs interface. The (in-plane) rigid rotation map of the lattice highlights how crystal defects and misfit stress nevertheless disturb the crystal lattice.

A detailed HR-STEM investigation revealed the presence of micro-twin defects (a type of planar defects) inside both types of InAs NWs as shown in Figure S7. These defects do not show sensitive influence in the geometric phase analysis maps as seen in Figure S8. No clear evidence of threading dislocations (a type of linear defects) was observed in the single-faceted NW, but at least one instance was clearly visible in the multi-faceted NW, as evidenced in Figures S7 and S8.

From the STEM images we see that whether an InAs NW is single- or multi-faceted is determined by the morphology of the underlying GaAs nanoridge. In the case of multi-faceted NWs, the GaAs nanoridge is underdeveloped, exhibiting roughness and morphological irregularity. For a fixed growth time, the stage of development of the GaAs nanoridge is a function of its local growth rate, which in turn is dependent on the presence and proximity of neighbouring nanoridges, as described in ref. 6. Growth time can also be adjusted to independently control the faceting of the NW, regardless of whether it is grown in isolation or in a parallel array.

7. Other THz measurements on InAs nanowire receivers

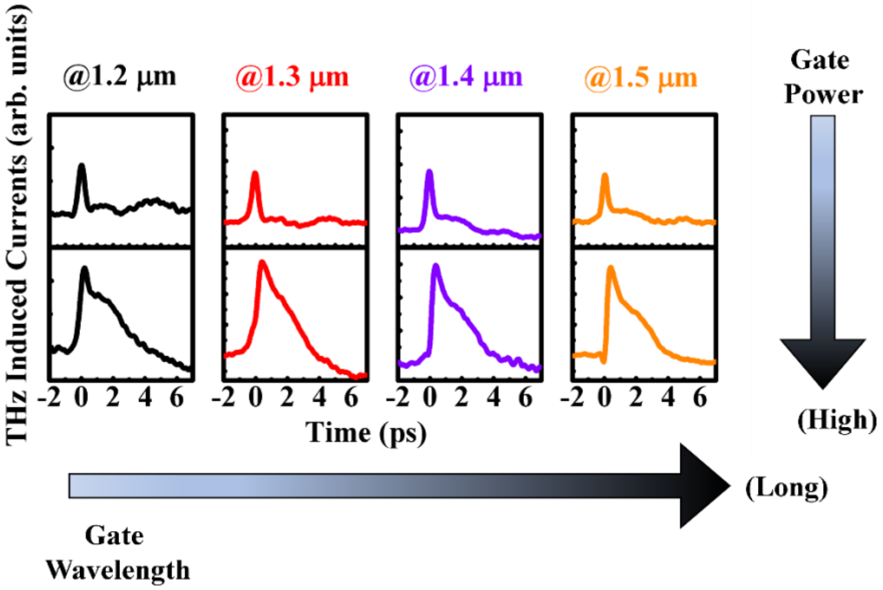

Figure S9. Wavelength and fluence investigation. Raw data of THz responses obtained from a (single-faceted) 5NW receiver (with a gap size of 1 μm), optically gated with 1.2-μm, 1.3-μm, 1.4-μm, 1.5-μm laser pulses, respectively. Top row: low excitation fluence of 0.05 mJ/cm^2; Bottom row: low excitation fluence of 0.98 mJ/cm^2.

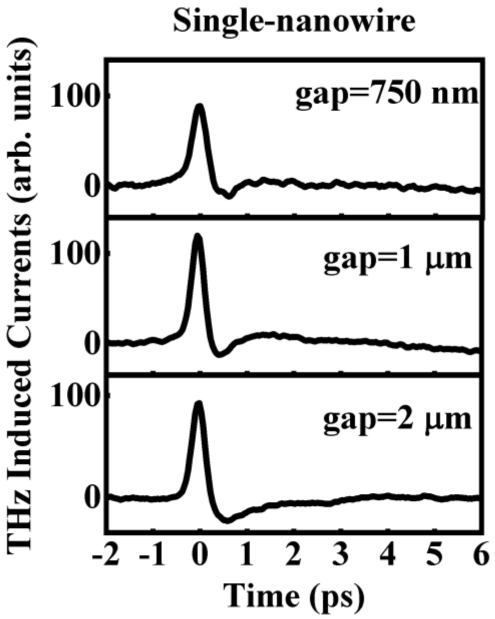

Figure S10. Antenna gap dependence. Comparison of raw data of THz responses obtained from 3 different 1NW receivers (optically gated at 1.55 μm under the fluence of 0.98 mJ/cm^2/pulse), in which the multi-faceted single NWs were grown with a nominal length of 6.75, 7 and 8 μm, respectively, and with corresponding antenna gap sizes of 0.75, 1 and 2 μm, respectively. Direct sampling is identified for all receivers independent of antenna gap size. The smaller gap size results in a smaller detection material volume (and thus less generated photocarriers in total) but more strongly enhanced THz field at the gap region, which results in similar level of current responses measured for all three receivers.

8. Side-facet tuning for multi-faceted InAs NWs

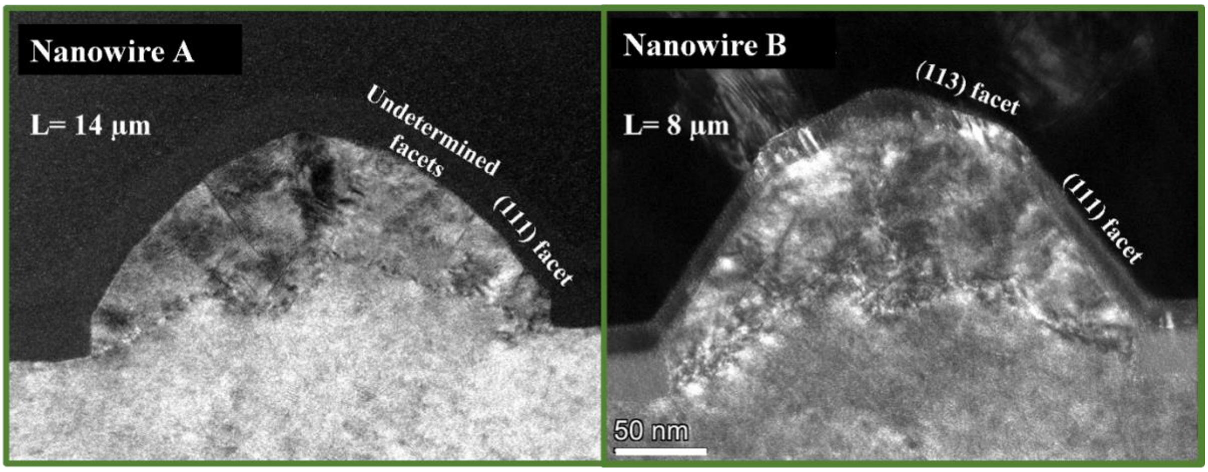

Figure S11. NW length-dependent faceting. Dark-field TEM images of cross sections of representative InAs NWs used in (multi-faceted) 1NW receivers. Left: when the nominal length of the NW is 14 µm. Right: when the nominal length of the NW is 8 µm.

References

1 Castro-Camus, E. *et al.* Photoconductive response correction for detectors of terahertz radiation. *Journal of Applied Physics* **104** (2008).
2 Peng, K. *et al.* Broadband Phase-Sensitive Single InP Nanowire Photoconductive Terahertz Detectors. *Nano Letters* **16**, 4925-4931 (2016).
3 Sun, M. H. *et al.* Removal of Surface States and Recovery of Band-Edge Emission in InAs Nanowires through Surface Passivation. *Nano Letters* **12**, 3378-3384 (2012).
4 Joyce, H. J. *et al.* Electronic properties of GaAs, InAs and InP nanowires studied by terahertz spectroscopy. *Nanotechnology* **24**, 214006 (2013).
5 Hÿtch, M. J., Snoeck, E. & Kilaas, R. Quantitative measurement of displacement and strain fields from HREM micrographs. *Ultramicroscopy* **74**, 131-146 (1998).
6 Dede, D. *et al.* Selective area epitaxy of GaAs: the unintuitive role of feature size and pitch. *Nanotechnology* **33**, 485604 (2022).
